# Appraising the Causal Role of Risk Factors in Coronary Artery Disease and Stroke: A Systematic Review of Mendelian Randomization Studies

**DOI:** 10.1161/JAHA.122.029040

**Published:** 2023-10-17

**Authors:** Andrea N. Georgiou, Loukas Zagkos, Georgios Markozannes, Christos V. Chalitsios, Alexandros G. Asimakopoulos, Wei Xu, Lijuan Wang, Ines Mesa‐Eguiagaray, Xuan Zhou, Eleni M. Loizidou, Nikolaos Kretsavos, Evropi Theodoratou, Dipender Gill, Stephen Burgess, Evangelos Evangelou, Konstantinos K. Tsilidis, Ioanna Tzoulaki

**Affiliations:** ^1^ Department of Hygiene and Epidemiology University of Ioannina School of Medicine Ioannina Greece; ^2^ Department of Epidemiology and Biostatistics School of Public Health, Imperial College London London UK; ^3^ Centre for Global Health, Usher Institute The University of Edinburgh Edinburgh UK; ^4^ Biobank Cyprus Center of Excellence in Biobanking and Biomedical Research University of Cyprus Nicosia Cyprus; ^5^ Cancer Research UK Edinburgh Centre, Institute of Genetics and Cancer The University of Edinburgh Edinburgh UK; ^6^ Medical Research Council Biostatistics Unit University of Cambridge Cambridge UK; ^7^ Cardiovascular Epidemiology Unit University of Cambridge Cambridge UK; ^8^ Department of Biomedical Research, Institute of Molecular Biology and Biotechnology Foundation for Research and Technology‐Hellas Ioannina Greece; ^9^ Centre for Systems Biology, Biomedical Research Foundation Academy of Athens Athens Greece

**Keywords:** cardiovascular disease, evidence grading, Mendelian randomizationsystematic review, Cardiovascular Disease, Epidemiology, Primary Prevention, Risk Factors, Genetics

## Abstract

**BACKGROUND:**

Mendelian randomization (MR) offers a powerful approach to study potential causal associations between exposures and health outcomes by using genetic variants associated with an exposure as instrumental variables. In this systematic review, we aimed to summarize previous MR studies and to evaluate the evidence for causality for a broad range of exposures in relation to coronary artery disease and stroke.

**METHODS AND RESULTS:**

MR studies investigating the association of any genetically predicted exposure with coronary artery disease or stroke were identified. Studies were classified into 4 categories built on the significance of the main MR analysis results and its concordance with sensitivity analyses, namely, robust, probable, suggestive, and insufficient. Studies reporting associations that did not perform any sensitivity analysis were classified as nonevaluable. We identified 2725 associations eligible for evaluation, examining 535 distinct exposures. Of them, 141 were classified as robust, 353 as probable, 110 as suggestive, and 926 had insufficient evidence. The most robust associations were observed for anthropometric traits, lipids, and lipoproteins and type 2 diabetes with coronary artery; disease and clinical measurements with coronary artery disease and stroke; and thrombotic factors with stroke.

**CONCLUSIONS:**

Despite the large number of studies that have been conducted, only a limited number of associations were supported by robust evidence. Approximately half of the studies reporting associations presented an MR sensitivity analysis along with the main analysis that further supported the causality of associations. Future research should focus on more thorough assessments of sensitivity MR analyses and further assessments of mediation effects or nonlinearity of associations.

**Nonstandard Abbreviations and Acronyms**
IL6R: interleukin 6 receptor
IVW: inverse variance weighted
MR: Mendelian randomization

**CLINICAL PERSPECTIVE**

**What Is New?**

- Numerous Mendelian randomization studies have examined the potential causal associations between risk factors and coronary artery disease or stroke; robust evidence for causality has been shown for only a minority of them.
- The findings of this systematic review suggest that coronary artery disease and stroke share a somewhat different profile of robust associations with protective/risk factors; coronary artery disease was robustly associated with anthropometric traits and lipoproteins, whereas stroke was robustly associated with inflammatory biomarkers and thrombotic factors.

**What Are the Clinical Implications?**

- Future research should comply with reporting guidelines for Mendelian randomization studies and focus on more thorough assessments of sensitivity Mendelian randomization analyses as well as other methodologies, including investigations of mediation effects and nonlinearity of associations.
- Risk factors with robust support for causality should be further investigated to guide better cardiovascular disease prevention policies and treatment.

Cardiovascular disease (CVD), principally coronary artery disease (CAD) and stroke, is the leading cause of death globally and a major contributor to disability worldwide.^1^ A large body of research has concentrated on identifying risk factors for CAD and stroke since the early cardiovascular observational studies in 1950s.^2^ These studies were instrumental in establishing the so‐called conventional cardiovascular risk factors such as raised blood pressure, raised serum cholesterol, cigarette smoking, and diabetes. However, beyond these conventional risk factors, an ever‐expanding list of exposures and their associations with cardiovascular manifestations is being explored in the medical literature.

Despite the volume of research, the causality of associations between risk factors and cardiovascular outcomes remains inconclusive for the majority of exposures, as observational associations are hindered by confounding and reverse causation and evidence from randomized controlled trials (RCTs) is relatively scarce.^3^ The Mendelian randomization (MR) approach can potentially overcome some biases of traditional epidemiological research by using genetic variants robustly associated with the risk factor of interest and assessing whether these variants are associated with the outcome of interest. The MR method can address bias attributed to confounding because genetic variants are randomly allocated when alleles are passed from parents to offspring during meiosis. MR studies therefore can be thought as *randomly assigning* participants based on the presence of alleles, which influence the risk factors of interest, and subsequently investigate whether carriers of genetic variants associated with the risk factor have different disease risks compared with noncarriers. In addition, as genetic variants are acquired at birth and cannot be modified by the presence of disease, MR associations are not influenced by reverse causality. Because of these appealing properties and as genome‐wide association studies (GWAS) provide associations between numerous traits and risk factors, MR is increasingly becoming a popular method to study the potential causal associations between different exposures and cardiovascular outcomes.

In this study, we present the first effort to systematically collect and appraise MR studies investigating any risk factor in relation to CAD and stroke. Our aim was to present the breadth and depth of exposures studied, identify areas of research focus and highlight gaps, and appraise the current evidence supporting their causal role in developing CAD and stroke.

## Methods

The data and materials that support the findings of this study are available in Data S1. Institutional review committee approval and consent by participants were not required because the current study is based exclusively on summary‐level data from previously published studies.

### Search Strategy

**FIGURE 1:**
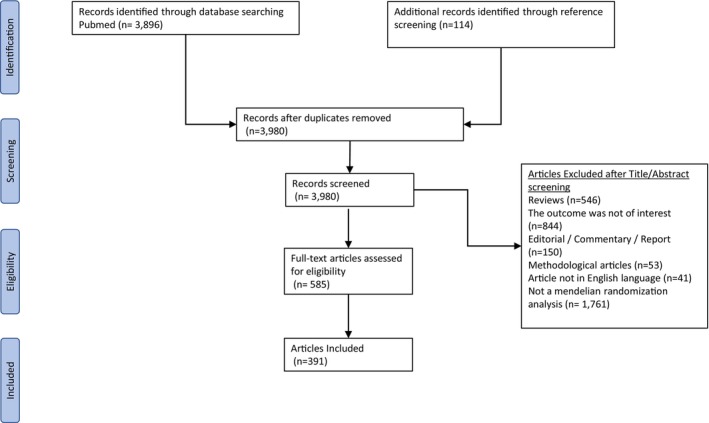
Flowchart of systematic literature search.

A systematic literature search was conducted independently by 2 researchers (A.N.G. and N.K.) on Medline (via PubMed) from inception to May 2022 for the identification of studies using the MR approach investigating causal risk factors for CAD or stroke. The following algorithm was used: “(Mendelian Randomization OR Mendelian Randomisation or genetic instrument) AND (Cardiovascular OR Stroke OR Coronary Heart OR Coronary Artery OR Myocardial Infarction).” We also screened the references of relevant reviews and the references of the included studies. The screening process in shown in Figure 1. The inclusion and exclusion criteria are described in detail in Data S1.

### Data Extraction

Data extraction was performed independently by 4 investigators (A.N.G., L.Z., C.V.C., A.G.A., E.M.L., L.W.) and independently double‐checked by 2 additional investigators (W.X., I.M.‐E.). From each eligible article, we recorded the first author, year of publication, the examined risk factors (exposure) and corresponding outcomes, the sample size for each exposure and outcome, the exposure and outcome population ancestry and source (ie, name of consortium), information for genetic variants modeled as instruments (*P* value threshold, threshold for linkage disequilibrium, biological relevance, power, percentage of variance explained by the instruments), MR design, main MR analysis used (ie, inverse variance weighted [IVW], maximum likelihood, Wald ratio, 2‐stage least squares), and the effect size (odds ratio [OR]) and the corresponding 95% CI and *P* value. We further extracted information on a number of sensitivity MR methods whenever these were performed and reported, for example, MR‐Egger, weighted median, and MR‐Pleiotropy Residual Sum and Outlier (PRESSO), as well as multivariable MR and all items included in the Strengthening the Reporting of Mendelian Randomization Studies checklist.^4^, ^5^ Outcomes included CAD (including myocardial infarction) and stroke. Stroke included overall stroke and main subtypes of stroke (eg, ischemic stroke, including small vessel ischemic stroke, large vessel ischemic stroke, and cardioembolic stroke as well as hemorrhagic stroke). (Further details on data extraction can be found in Data S2.)

### Data Synthesis and Evaluation of Robustness

Based on the extracted information, we presented the basic characteristics of the identified MR analyses. Main findings were categorized by risk factor and risk factor categories. The robustness of the evidence (robust, probable, suggestive, and insufficient evidence) was assessed through a priori defined criteria (Figure S1, Data S3)^6^ based on previous recommendations.^7^ We grouped MR studies into polygenic (*trans*) MR studies (studies that use variants from multiple regions of the genome associated with the risk factor of interest) and monogenic (*cis*) MR studies (studies using biological knowledge and variants from a single‐gene region associated with the risk factor of interest). For example, an MR analysis for CRP (C‐reactive protein) can be monogenic, and therefore conducted using variants in the *CRP* gene only, or polygenic, and therefore conducted using all independent genome‐wide significant variants associated with CRP.^7^

For polygenic MR studies, we based the evaluation on the results of the main MR analysis and the sensitivity analyses (eg, MR‐Egger, weighted median, MR‐PRESSO). The sensitivity analyses are used to check potential violations of the assumptions of the MR methodology. Evidence for causality was therefore considered stronger when a sensitivity analysis was reported and was supportive of the main analysis findings as MR investigations that do not perform ≥1 sensitivity method may be viewed as having incomplete evidence. Specifically, the associations were considered as having robust evidence for causality when all methods had concordant direction of effect estimates and both the main analysis and at least 1 sensitivity analysis achieved statistical significance (*P*<0.05). When studies adjusted their results for multiple testing, we used the *P* value threshold after the adjustment to define statistical significance, otherwise we used a nominal significance level (*P*<0.05). When a *P* value was not reported for the main MR estimate, we calculated it using the effect size and standard error. Also, when studies also reported analyses excluding genetic variants with evidence of pleiotropy, we considered those as the main analysis as they account better for the MR assumptions. The term *robust* refers to evidence of causality for the studied associations, not the quality of the analysis. An association was supported by probable evidence for causality when at least 1 method (main or sensitivity analysis) achieved statistical significance and the direction of the effect estimate was concordant in all methods. Suggestive evidence for causality was achieved when at least 1 method had a statistically significant *P* value but the direction of the effect estimates differed between methods. Associations that presented nonsignificant *P* values for both the main analysis and sensitivity analyses were classified as insufficient evidence for causality. Polygenic MR studies that did not report any sensitivity analyses were nonevaluable based on the aforementioned grading scheme that focuses on evaluating the robustness for causality of the studied associations.

Monogenic MR studies included MR analyses examining only one single‐nucleotide polymorphism (SNP) or single‐gene regions to define the risk factor (instrumental variable of interest). Most of these studies could not perform sensitivity analyses as the number of genetic variants was small. We assessed the robustness of these results based on whether the authors also performed colocalization analysis.^8^ Colocalization assesses whether the same genetic variant (or variants) influences 2 traits and is useful when MR is based on a single‐gene region.^7^

Finally, we further assessed the reporting of all MR studies using the Strengthening the Reporting of Mendelian Randomization Studies guidelines.^4^, ^5^

All statistical analyses were done with R 4.1.0.

## Results

### Eligible Studies

The literature search yielded 3980 articles of which 586 were evaluated in full text, and of them, 391 publications were deemed eligible (see full list in Tables S1 and S2, respectively). The majority of studies were published from 2018 onward (Figure S2).

### Description of Study Characteristics

**FIGURE 2:**
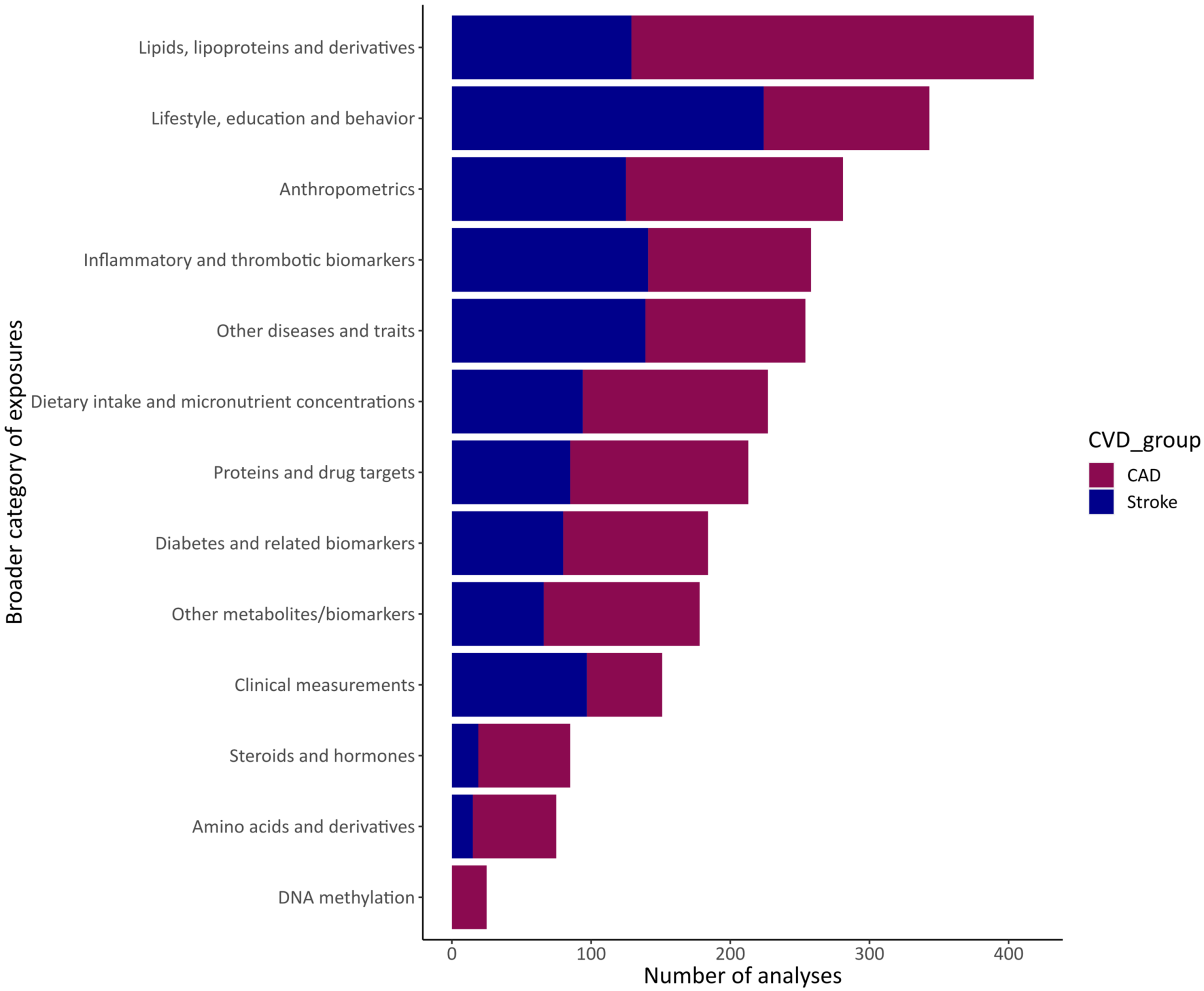
Number of Mendelian randomization associations extracted from eligible publications according to different exposure categories for coronary artery disease (CAD) and stroke.

Of 391 MR publications, 317 studied CAD as the outcome of interest, 175 stroke, and 102 both outcomes. Overall, the 391 publications included 2725 different MR analyses examining 535 unique exposures, 482 in relation to CAD and 268 in relation to stroke, covering a broad range of biomarkers, physical measurements, traits, and diseases (Figure 2, Table S3). Many risk factors have been examined in multiple MR publications (Table S4), and the most commonly studied risk factors were low‐density lipoprotein cholesterol for CAD (26 articles) and body mass index for stroke (10 articles).

There were 2122 polygenic MR analyses and 596 monogenic MR analyses. The median number of SNPs used to genetically predict the risk factor of interest in polygenic MR studies was 13, ranging from 2 to 3188 SNPs (Table S5). The median sample size for the exposure genetic analysis was 81 807 (with the smallest being 272 for phospholipase A2 and the largest 1 887 658 for COVID‐19 severity). GWAS summary statistics for the exposures were derived from European (87.5%) and multiethnic (9.9%) ancestry populations. For the outcomes, 65.9% of the associations were derived from European and 32.5% from multiethnic populations. The vast majority of MR analyses used or included CARDIoGRAMplusC4D (Coronary Artery Disease Genomewide Replication and Meta‐Analysis Plus the Coronary Artery Disease Genetics consortium) when CAD was the outcome of interest (822 of 1478 MR analyses) and MEGASTROKE consortium when stroke was the outcome of interest (914 of 1214 MR analyses). Only 65 MR analyses were based on 1‐sample MR designs.

### Evaluation of the Robustness of Causal Associations

Table S6 lists the main characteristics of each eligible MR analysis and its subsequent grading category based on the robustness of evidence, whereas Table S7 summarizes the grading categories. Of the 2122 polygenic MR associations, 20 analyses were based on 2 genetic variants (in different gene regions) and sensitivity analysis could not be performed. From the remaining 2102 MR analyses, 1479 (70%) presented results on both the main and at least 1 sensitivity analysis and were eligible for evaluation. IVW was the main analysis in the majority of associations examined (N=1931, 92%). Of the 1530 associations reporting main and sensitivity analyses, we found 141 robust associations (median N_SNPs_=110), 353 probable associations (median N_SNPs_=71), 110 suggestive associations (median N_SNPs_=73), and 926 associations with insufficient information (median N_SNPs_=21).

Overall, 276 MR analyses reported multivariable MR, which examines multiple risk factors (exposures) simultaneously and estimates the independent causal effect of each of the risk factors. Genetically predicted body mass index, smoking, and lipid levels were common risk factors adjusted in multivariable MR.

There were also 596 monogenic MR associations examining genetic variants within a single‐gene region as instrumental variables for the risk factor of interest. Of them, 447 were based on a single genetic variant analysis only (Figure S3). Among the 596 associations, 219 reported statistically significant results in the main analysis. Of them, 24 analyses performed colocalization analyses with the outcome of interest, of which 2 found evidence for colocalization between the risk factor and the outcome (ICA1L [islet cell autoantigen 1‐like protein] for stroke and NK3R [neurokinin 3 receptor] for CHD).

**FIGURE 3:**
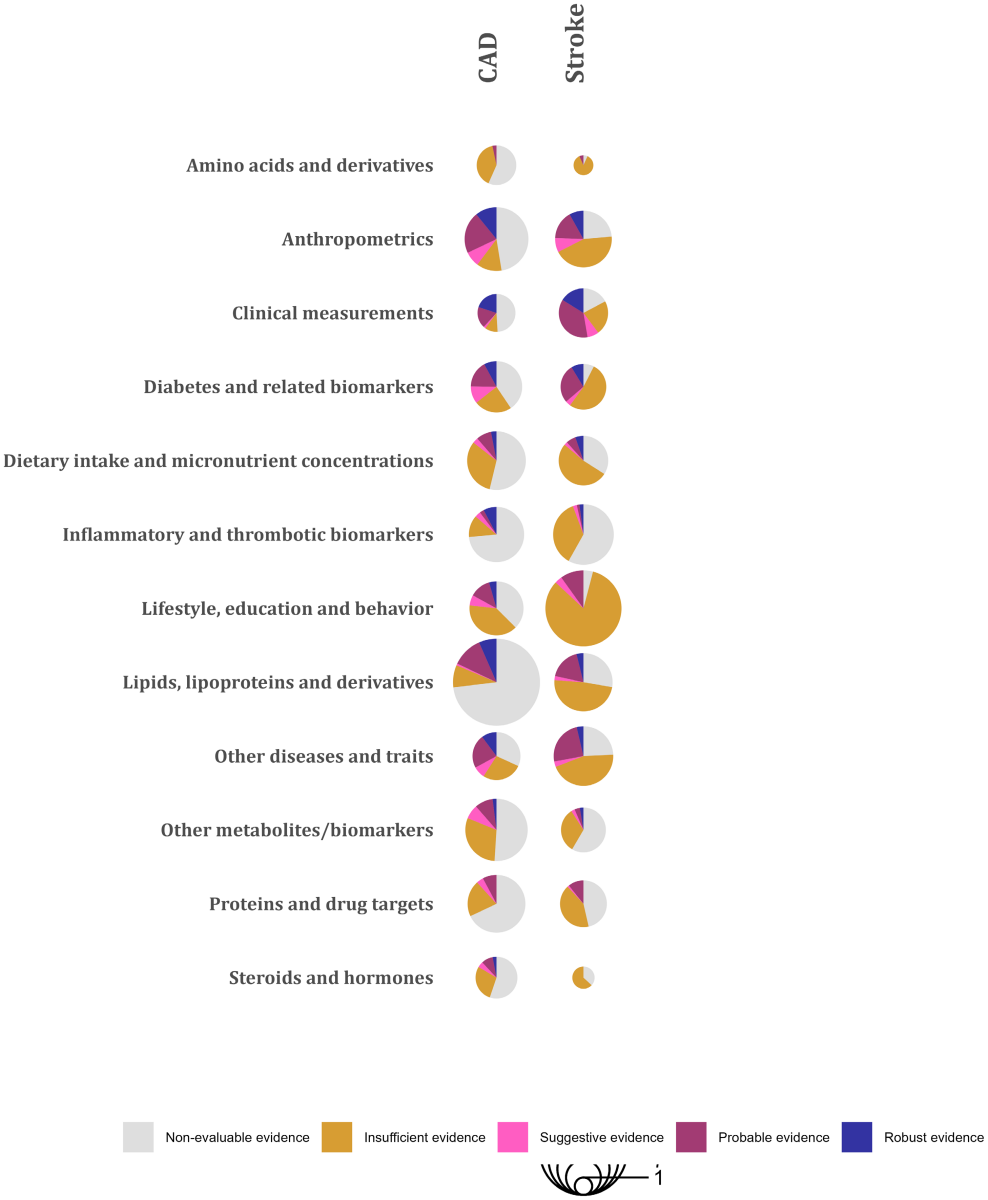
Evidence map for eligible Mendelian randomization studies per exposure category for CAD and stroke. DNA methylation was not included in the diagram because of the limited number of analyses for the specific exposure category. Nonevaluable evidence level includes associations for which a sensitivity analysis was not feasible (eg, single genetic variant analyses). CAD indicates coronary artery disease; IVW, inverse variance weighted; OR, odds ratio; Ref, reference number; SNP, single‐nucleotide polymorphism; and WM, weighted median.

**FIGURE 4:**
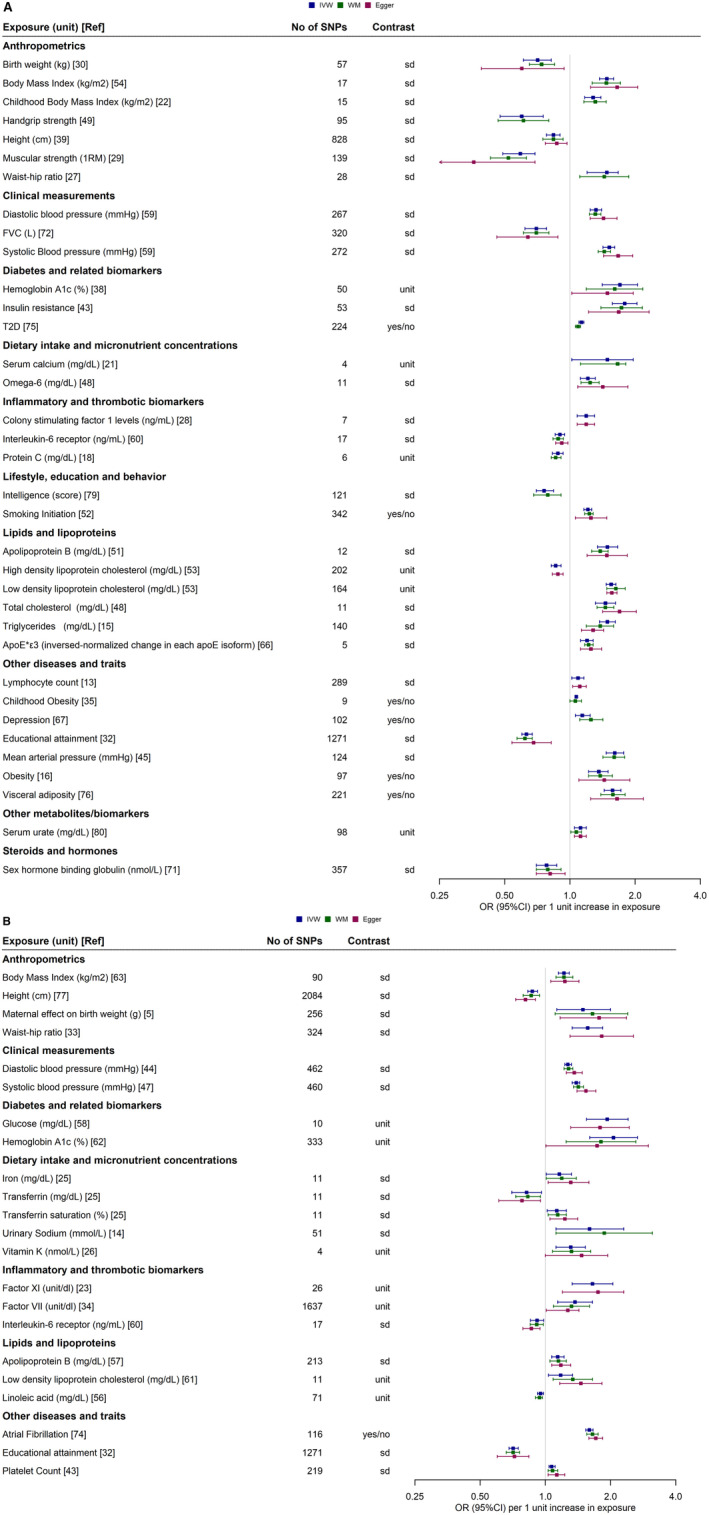
Forest plot showing the identified robust associations between exposures and CAD (A) and stroke (B). When >1 study exhibited a robust association with CAD or stroke, the most recent study with the largest sample size (exposure) was selected. The number in square brackets corresponds to the reference that examined the relevant exposure. ApoE indicates apolipoprotein E; CAD, coronary artery disease; CIS, cardioembolic ischemic stroke; FVC, forced vital capacity; IS, ischemic stroke; IVW, inverse variance weighted; LVIS, large vessel ischemic stroke; MI, myocardial infarction; OR, odds ratio; Ref, reference number; RM, repetition maximum; SNP, single‐nucleotide polymorphism; and WM, weighted median.

A graphical overview of the robustness of the evidence per exposure category and CVD group is presented in Figure 3. The exposure category with the most robust associations was anthropometry (N=28), followed by lipids and lipoproteins (N=24). There were 141 robust polygenic MR associations pertaining to 53 different risk factors as illustrated in detail in Table S8 and Figure 4 (35 risk factors for CAD and 22 risk factors for stroke).^9^, ^10^, ^11^, ^12^, ^13^, ^14^, ^15^, ^16^, ^17^, ^18^, ^19^, ^20^, ^21^, ^22^, ^23^, ^24^, ^25^, ^26^, ^27^, ^28^, ^29^, ^30^, ^31^, ^32^, ^33^, ^34^, ^35^, ^36^, ^37^, ^38^, ^39^, ^40^, ^41^, ^42^, ^43^, ^44^, ^45^, ^46^, ^47^, ^48^, ^49^, ^50^, ^51^, ^52^, ^53^, ^54^, ^55^, ^56^, ^57^, ^58^, ^59^, ^60^, ^61^, ^62^, ^63^, ^64^, ^65^, ^66^, ^67^, ^68^, ^69^, ^70^, ^71^, ^72^, ^73^, ^74^, ^75^, ^76^ Almost all studies that showed robust evidence of association between an exposure and CAD or stroke had a good reporting score for Strengthening the Reporting of Mendelian Randomization Studies reporting, with the exception of 3 studies (Table S9).^60^, ^71^, ^73^ Apart from conventional cardiovascular risk factors such as blood pressure traits, cholesterol levels, type 2 diabetes, obesity, and smoking, robust positive associations were observed between genetically predicted calcium (OR_IVW_, 1.66 [95% CI, 1.12–1.81]), lymphocyte count (OR_IVW_, 1.09 [95% CI, 1.02–1.16]), colony stimulating factor 1 (OR_IVW_, 1.19 [95% CI, 1.08–1.30]), and omega 6 fatty acid levels (OR_IVW_, 1.21 [95% CI, 1.12–1.31]) with CAD. Protective (inverse) associations were also observed for genetically predicted height (OR_IVW_, 0.84 [95% CI, 0.78–0.90]), forced vital capacity (OR_IVW_, 0.64 [95% CI, 0.46–0.88]), sex hormone binding globulin (OR_IVW_, 0.79 [95% CI, 0.7–0.91]), and IL6R (interleukin 6 receptor; OR_IVW_, 0.9 [95% CI, 0.85–0.95]). For stroke, robust associations were observed for genetic predisposition to several thrombotic factors, apolipoprotein B (OR_IVW_, 1.14 [95% CI, 1.07–1.22]), urinary sodium excretion (OR_IVW_, 1.6 [95% CI, 1.12–2.3]) (positive), and IL6R (OR_IVW_, 0.92 [95% CI, 0.85–0.98]) and transferrin (OR_IVW_, 0.82 [95% CI, 0.70–0.96]) (inverse).

Of the robust associations, 24 reported multivariable MR analyses adjusting for potential mediators. Of these, 2 associations were attenuated to the null: linoleic acid and stroke after adjusting for low‐density lipoprotein cholesterol and forced vital capacity, and CHD after adjusting for height. The remaining multivariable analyses attenuated the estimates, suggesting different levels of mediation; however, statistical significance was retained (Table S10).

## Discussion

In this systematic review, we summarized the evidence for associations between genetic predisposition to 535 risk factors and CAD or stroke examined in 391 publications covering 2725 MR associations. Using a set of predefined criteria, we found robust evidence for causality between 35 distinct risk factors and CAD and between 22 risk factors and stroke. For CAD, these included the well‐established cardiovascular risk factors such as blood pressure, type 2 diabetes, obesity, smoking, and cholesterol levels but also anthropometry and physical measurements (height, birth weight, muscular strength, and forced vital capacity) and several biomarkers (leucocyte count, serum calcium, IL6R signaling, protein C, omega 6 fatty acid levels, sex hormone binding globulin). Stroke showed a somewhat different profile of associations, with evidence for causal effect for thrombotic risk factors (factor VII, factor XI, platelet count, vitamin K), iron and inflammatory biomarkers (iron, transferrin, transferrin saturation, IL6R), and blood pressure.

This large body of published MR analyses highlighted several reporting limitations also observed in a previous systematic review of MR studies on cancer outcomes.^6^ Approximately half of the associations included sensitivity analyses, which are important to assess the assumptions of the method and therefore the robustness of the results. The lack of sensitivity analyses was often because studies were published early before the availability of MR sensitivity methods or because they were monogenic (single‐gene) or single variant MR studies where sensitivity analyses were not feasible because of the small number of instrumental variables. In the latter case of MR studies, colocalization can be used to investigate whether the exposure and the outcome share a causal variant in the genetic region, but it was rarely performed in the examined MR studies. Again, this may be attributed to the fact that colocalization was only suggested recently as an additional method to support monogenic MR investigations and a large proportion of these studies were published earlier. Genetic variants typically explain only a small proportion of the variation in the relevant exposure of interest, and as a result low statistical power is common in MR studies. The MR studies examined here rarely reported power estimates or the variance explained by instrumental variables, and therefore it was difficult to conclude whether nonsignificant associations were true null findings.^77^ The recent publication of the MR reporting guidelines (Strengthening the Reporting of Mendelian Randomization Studies) statement should improve the reporting standards of MR studies and further enhance the robustness and interpretability of MR findings.^5^ Finally, MR investigations are dependent on the primary GWAS sources, the quality of which was not assessed in this work. However, underlying GWAS quality is unlikely to lead to false positive results.

A considerable proportion of the studies provided supporting evidence for causal associations between the so‐called conventional CVD risk factors and CVD events. We identified studies with robust evidence for causal associations between genetically predicted low‐density lipoprotein cholesterol, high‐density lipoprotein cholesterol, triglycerides, apolipoprotein B, blood pressure, type 2 diabetes, and glycemic traits such as hemoglobin A1c and insulin resistance with CAD and stroke. This supports the extensive evidence from traditional epidemiological studies, experimental studies, and RCTs examining these risk factors. However, MR provided additional valuable information such as examination of comparative effects between correlated risk factors, estimation of nonlinear effects, and interactions with other factors. For example, multivariable MR analyses on several lipids and lipoproteins highlighted the central role of apolipoprotein B compared with other lipids in ischemic stroke.^53^ Similarly, the MR paradigm generated evidence supporting an effect of midlife blood pressure on later life CAD risk independent of later life blood pressure.^41^

Measures of anthropometry have also been extensively studied in the MR context in relation to CAD and stroke. Beyond body mass index, which showed robust causal associations with CAD and stroke, higher height was also highlighted as a potentially causal risk factor for CAD. Genetically predicted height also mediated at least partly the association between lung function measured by forced vital capacity and CHD and stroke. Although several observational studies have reported a protective role of short height for CVD, the magnitude of this association has been controversial,^78^ and the mechanisms underlying this inverse association are not well understood.^79^ One proposed explanation is that shorter individuals have on average smaller vessel diameters, which can lead to increased arterial occlusive events.^80^, ^81^ There was also both robust and probable evidence for a protective association between higher birth weight and CAD and stroke, respectively, supporting the fetal developmental origins of CVD.^26^, ^82^ Interestingly, further investigation into the fetal or maternal components of instrumental effects on birth weight showed robust evidence between lower birth weight, by maternal rather than fetal genome, and stroke and its subtypes in later life.^51^

Lifestyle is an important area of MR research in CVD as RCTs are often inappropriate or unfeasible, and evidence stemming from MR is vital to support causality. Smoking behavior showed robust evidence for a causal association with CVD in agreement with overwhelming evidence from observational epidemiology.^83^ Coffee, alcohol consumption, and sleep duration also showed probable associations.^48^, ^84^, ^85^, ^86^, ^87^, ^88^, ^89^ Observational epidemiology has often suggested a possible protective effect of moderate alcohol consumption on CVD. Dose–response MR analyses did not support this conclusion, but they found evidence of a dose–response relationship between alcohol and risk of stroke.^90^ Educational attainment was reported to have a protective role for both CAD and stroke, exhibiting robust evidence of association in MR studies.^15^, ^28^, ^33^ Traditional observational studies and MR mediation analyses have shown that body mass index, systolic blood pressure, and smoking behavior mediate a substantial proportion of the protective effect of education on the risk of CVD outcomes.^91^, ^92^ Despite the research interest on diet and CVD, there were few robust or probable associations between nutrients or dietary traits and CVD outcomes. This is partly expected because of the low power of genetically predicted nutrients and other dietary variables (few SNPs available to instrument dietary traits) and the small heritable components of many dietary traits both leading to underpowered MR studies.

Many MR analyses concentrate on the causal association between biomarker levels and CVD to identify novel treatment targets for the disease. Of them, genetically predicted plasma sIL6R (soluble IL6R), an IL6 (interleukin 6) signaling biomarker, showed robust evidence for an inverse association with CAD^36^ and stroke,^60^ supporting a key role of inflammation in CVD, which also has supportive RCT evidence.^93^ Thrombotic factors are implicated in the coagulation cascade and along with inflammatory factors are contributing to the suppression of a pathogen entering in the host, a mechanism termed as immunothrombosis. The aberrant activation of immunothrombosis has been associated with increased risk for myocardial infarction, stroke and venous thromboembolism.^94^ This association is supported by MR evidence. A robust positive association was observed between vitamin K and large vessel stroke as well as between two enzymes of the coagulation cascade (ie, factor XI and factor VII) and ischemic stroke.^19^, ^22^, ^30^ In contrast to thrombotic factors, Protein C, also known as factor XIX, a zymogen that inactivates thrombotic enzymes, showed evidence for an inverse causal effect with CAD.^14^ In concordance with meta‐analyses of RCTs for calcium supplementation,^95^, ^96^ MR evidence supported a causal association between higher serum calcium levels and increased CAD risk.^17^, ^97^, ^98^, ^99^ Circulating calcium levels are thought to increase CAD risk through vascular calcification^100^, ^101^ or via the upregulation of the coagulation pathway which in turn is associated with CVD risk.^102^ Finally, iron, ferritin, and transferrin saturation, biomarkers of iron metabolism and intake,^103^ showed robust positive causal associations with risk of stroke in MR analyses and this effect was suggested to be driven by an increased risk of cardioembolic stroke.^21^ The latter, along with the absence of an association with CAD, may indicate that the effect of iron on stroke is through thrombus formation rather than atherosclerosis.^104^

## Strengths and Limitations

In the current systematic review, we summarized all previously published MR studies for all genetically determined exposures and their association with CVD risk. A clear categorization scheme and evaluation criteria were applied, to further examine the robustness credibility of the resulting associations. Other efforts to summarize the evidence of MR analyses on CVD risk have been performed in the past. However, they were either limited to specific exposures,^105^ or used a more narrative approach of presenting and assessing the MR results,^106^ while none performed a formal evaluation of the evidence. However, some limitations exist, which need to be acknowledged. Some relevant MR studies may have been missed through our search strategy, especially if the MR analysis was not the primary focus but only a supplementary analysis, which seems to be increasingly common in recent GWAS. In the absence of comprehensive MR guidelines, we based our evaluation of the evidence of causality adapting a set of previously proposed criteria. This approach did not allow us to investigate MR studies presenting only main analysis without sensitivity analyses. Sensitivity analyses increase the credibility of the findings as they test various MR assumptions. However, many studies did not present those as they were published earlier before such analyses were introduced in the literature or were based on monogenic associations with a small number of SNPs which did not allow sensitivity analyses. For the latter associations we based our evaluation on availability of colocalization analysis which was again introduced only recently. Therefore, the evaluation criteria for this systematic review were designed mainly for the assessment of the evidence that resulted from the MR analyses and not for the assessment of the quality of the studies. Although many studies included instrumental variables from the largest available GWAS for the exposure traits, the SNPs explained a small percentage of the variance and therefore some studies were underpowered. Finally, information on statistical power of the instrument was often not reported, and therefore the grading scheme used could not distinguish between MR analyses with robust evidence of lack of association or MR analyses which did not present an association due to lack of power.

## Conclusions

MR studies have contributed a large body of evidence supporting the causal association between risk factors and CVD. Although many studies concentrated on CVD risk factors known to be causally associated with CVD through RCTs, MR provided further confirmation of previous associations and supported evidence for potentially novel causal risk factors. Despite the plethora of MR investigations in CVD, the highlighted associations with robust evidence for causality were modest. Those risk factors concentrated around conventional risk factors for CVD, inflammation and thrombotic factors, and indices of anthropometry and showed a large overlap between risk factors for CAD and stroke, as well as highlighted the different risk factor profiles between stroke subtypes. As GWAS investigations of exposures become larger, novel exposures are measured in epidemiological settings, and novel MR methodologies are published, the contribution of MR in establishing causal associations and prioritizing RCT is expected to grow further.

## Sources of Funding

The project is cofinanced by the European Regional Development Fund of the European Union and Greek National Funds through (1) the Operational Program Competitiveness, Entrepreneurship and Innovation (EPAnEK), National Strategic Reference Framework (NSRF) 2014–2020 (project code MIS: OΠΣ 5047228) and (2) the Operational Programme Epirus 2014–2020 of the Prefecture of Epirus (project code Management Information System (MIS): HΠ1AB‐0028180). The authors acknowledge generous support by the National Institutes of Health (R01HL133932 and R01HL111362), Medical Research Council and National Institute for Health Research (MC_PC_12025), the Medical Research Council (MRC) Centre for Environment and Health (MR/S019669/1), and UK Dementia Research Institute (MC_PC_17114), which is supported by the Medical Research Council, the Alzheimer's Society, and Alzheimer's Research UK.

## Disclosures

None.

## Supporting information

Data S1–S3
Figures S1–S3

Tables S1–S10
References 107–139
