# Appraising the causal role of risk factors in coronary artery disease and stroke: A systematic review of Mendelian Randomization studies

**DOI:** 10.1161/JAHA.122.029040

**Published:** 2023-10-07

**Authors:** Andrea N Georgiou, Loukas Zagkos, Georgios Markozannes, Christos V. Chalitsios, Alexandros Georgios Asimakopoulos, Wei Xu, Lijuan Wang, Ines Mesa-Eguiagaray, Xuan Zhou, Eleni M Loizidou, Nikolaos Kretsavos, Evropi Theodoratou, Dipender Gill, Stephen Burgess, Evangelos Evangelou, Konstantinos K Tsilidis, Ioanna Tzoulaki

**Affiliations:** 1 Department of Hygiene and Epidemiology, University of Ioannina School of Medicine, Ioannina, Greece; 2 Department of Epidemiology and Biostatistics, School of Public Health, Imperial College London, London, United Kingdom; 3 Centre for Global Health, Usher Institute, The University of Edinburgh, Edinburgh, UK; 4 CRUK Edinburgh Centre, Institute of Genetics and Cancer, The University of Edinburgh, Edinburgh, UK; 5 Biobank.cy Center of Excellence in Biobanking and Biomedical Research, University of Cyprus; 6 Medical Research Council Biostatistics Unit, University of Cambridge, Cambridge, UK; 7 Cardiovascular Epidemiology Unit, University of Cambridge, Cambridge, UK; 8 Department of Biomedical Research, Institute of Molecular Biology and Biotechnology, Foundation for Research and Technology-Hellas, Ioannina, Greece; 9 BHF Centre of Research Excellence, Imperial College London, London, UK

**Keywords:** Mendelian randomization, systematic review, cardiovascular disease, evidence grading

## Abstract

**Background:**

Mendelian randomization (MR) offers a powerful approach to study potential causal associations between exposures and health outcomes, by using genetic variants associated with an exposure as instrumental variables. In this systematic review, we aimed to summarize previous MR studies and to evaluate the evidence for causality for a broad range of exposures in relation to coronary artery disease (CAD) and stroke.

**Methods:**

MR studies investigating the association of any genetically predicted exposure with CAD or stroke were identified in Pubmed. Studies were classified into four categories, namely *robust, probable, suggestive* and *insufficient*, built on the significance of the main MR analysis results and its concordance with sensitivity analyses (MR-Egger, weighed median and MR-PRESSO). Associations that did not perform any sensitivity analysis were classified as *non-evaluable*.

**Findings:**

We identified 2,718 associations eligible for evaluation, examining 535 distinct exposures. Of them, 138 were classified as *robust*, 347 as *probable*, 109 as *suggestive* and 886 had *insufficient* evidence. The most prominent *robust* associations were observed for anthropometric traits (i.e., body mass index, height, waist to hip ratio and birth weight) and lipids and lipoproteins (i.e., low- and high-density lipoproteins, triglycerides) and type 2 diabetes with CAD, clinical measurements (i.e., systolic and diastolic blood pressure) with CAD and stroke, and thrombotic factors (i.e., factors XI and VII, iron and vitamin K) with stroke.

**Conclusion:**

Despite the large number of studies that have been conducted, only a limited number of associations were supported by robust evidence. About half of the associations presented a MR sensitivity analysis along with the main analysis which further supported the causality of associations. Future research should focus on more thorough assessment of sensitivity MR analyses and assessment of mediation effects and nonlinearity in associations.

## Introduction

Cardiovascular disease (CVD), principally coronary artery disease (CAD) and stroke, is the leading cause of death globally and a major contributor to disability worldwide[1]. A large body of research has concentrated on identifying risk factors for CAD and stroke since the early cardiovascular observational studies in 1950s [2]. These studies were instrumental in establishing the so-called conventional cardiovascular risk factors such as raised blood pressure, raised serum cholesterol, cigarette smoking and diabetes mellitus. However, beyond these conventional risk factors, an ever-expanding list of exposures and their associations with cardiovascular manifestations is being explored in the medical literature.

Despite the volume of research, the causality of associations between risk factors and cardiovascular outcomes remains inconclusive for the majority of exposures, as observational associations are hindered by confounding and reverse causation and evidence from randomized controlled trials is relatively scarce [3]. The Mendelian randomization (MR) approach can potentially overcome some biases of traditional epidemiological research by using genetic variants robustly associated with the risk factor of interest and assessing whether these variants are associated with the outcome of interest. The MR method can address bias due to confounding because genetic variants are randomly allocated when alleles are passed from parents to offspring during meiosis. MR studies therefore can be thought as ‘randomizing’ participants based on the presence of alleles which influence the risk factors of interest and subsequently investigate whether carriers of genetic variants associated with the risk factor have different disease risk compared to non-carriers. Additionally, as genetic variants are acquired at birth and cannot be modified by the presence of disease, MR associations are not influenced by reverse causality. Due to these appealing properties and as GWAS studies provide associations between numerous traits and risk factors, MR is increasingly becoming a popular method to study the potential causal associations between different exposures and cardiovascular outcomes.

In this study we present the first effort to systematically collect and appraise MR studies investigating any risk factor in relation to CAD and stroke. Our aim was to present the breadth and depth of exposures studied, identify areas of research focus and highlight gaps, and to appraise the current evidence supporting their causal role in developing CAD and stroke.

## Methods

### Search Strategy

**Figure 1:**
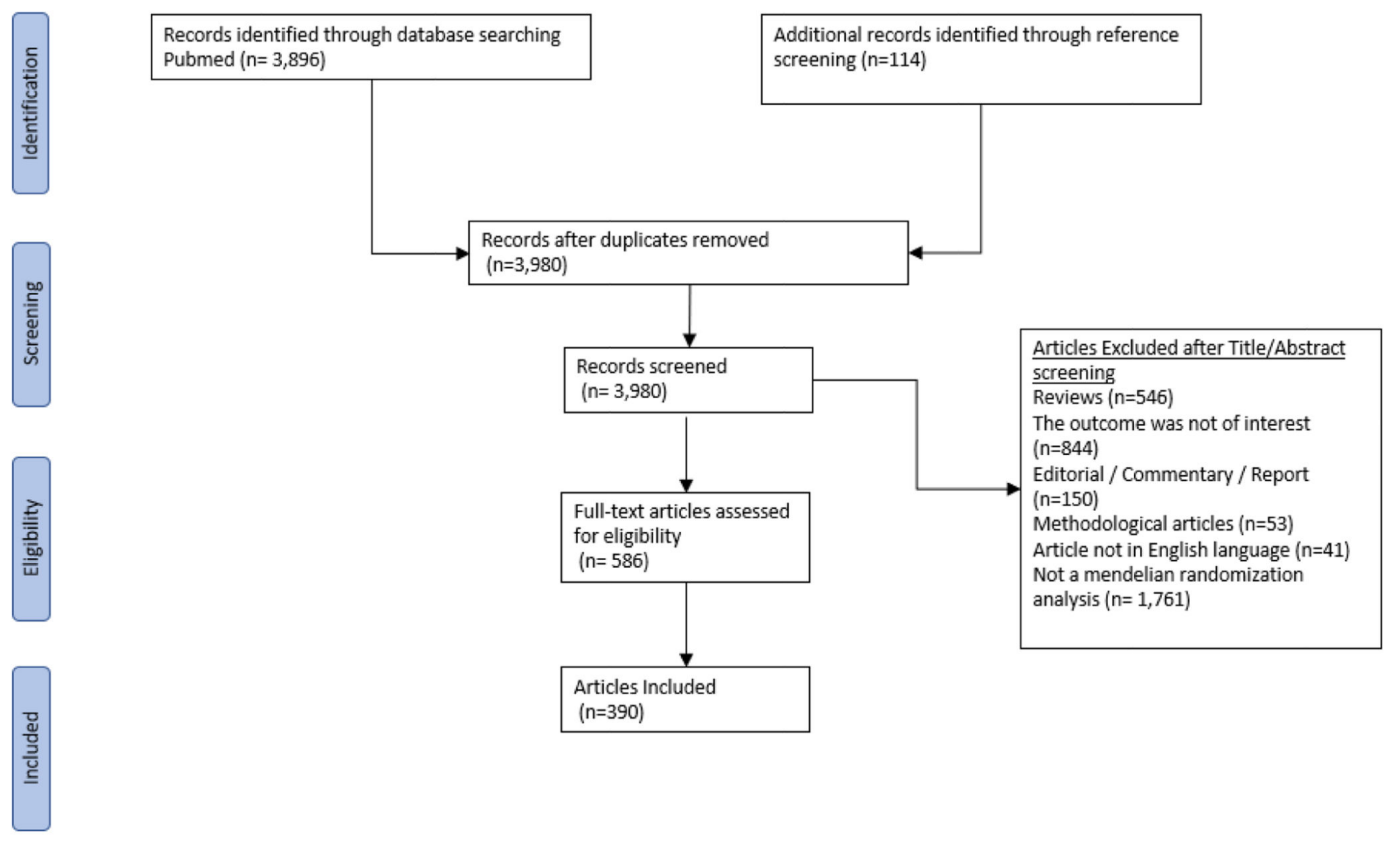
Flow chart of systematic literature search

A systematic literature search was conducted independently by two researchers (A.G and N.K) on Medline (via PubMed) from inception up to May 2022, for the identification of studies using the MR approach investigating causal risk factors for CAD and/or stroke. The following algorithm was used: “(Mendelian Randomization OR Mendelian Randomisation or genetic instrument) AND (Cardiovascular OR Stroke OR Coronary Heart OR Coronary Artery OR Myocardial Infarction)”. We also screened the references of relevant reviews and the references of the included studies. The screening process in shown in Figure 1.

### Data Extraction

Data extraction was performed independently by four investigators (AG, LZ, CVC, AGA, EL, LW), and independently double-checked by two additional investigators (WX, IME). From each eligible article, we recorded the first author, year of publication, the examined risk factors (exposure) and corresponding outcomes, the sample size for each exposure and outcome, the exposure and outcome population ancestry and source (i.e. name of consortium), information for genetic variants modelled as instruments (p-value threshold, threshold for linkage disequilibrium, biological relevance, power, percentage of variance explained by the instruments), MR design, main MR analysis used (i.e. inverse variance weighted (IVW), maximum likelihood, Wald ratio, two-stage least squares), the effect size (odds ratio) and the corresponding 95% confidence interval (CI) and p-value. We further extracted information on a number of sensitivity MR methods, whenever these were performed and reported e.g., MR-Egger, weighted median (WM), MR-PRESSO and also multivariable MR (MVMR) and all items included in the Strengthening the Reporting of Mendelian Randomization Studies (STROBE-MR) checklist [4, 5].

### Data synthesis and evaluation of robustness

Based on the extracted information, we presented the basic characteristics of the identified MR analyses. Main findings were categorized by risk factor and risk factor categories. The robustness of the evidence (*robust, probable, suggestive* and *insufficient* evidence) was assessed through a-priori defined criteria (Figure S1) [6] based on previous recommendations [7]. We grouped MR studies into polygenic MR studies (studies which use variants from multiple regions of the genome associated with the risk factor of interest) and monogenic MR studies (studies using biological knowledge and variants from a single gene region associated with the risk factor of interest). For example, a MR analysis for C-reactive protein (CRP) can be monogenic and therefore conducted using variants in the CRP gene only or polygenic and therefore conducted using all independent genome-wide significant variants associated with CRP [7].

For polygenic MR studies, we based the evaluation on the results of the main MR analysis and the sensitivity analyses (e.g., MR-Egger, WM, MRPRESSO). The sensitivity analyses are used to check potential violations of the assumptions of the MR methodology. Evidence for causality was therefore considered stronger when a sensitivity analysis was reported and was supportive of the main analysis findings as MR investigation that do not perform one or more sensitivity methods may be viewed as having incomplete evidence. More specifically, the associations were considered as having *robust* evidence for causality when all methods had concordant direction of effect estimates and both the main analysis and at least one sensitivity analysis achieved statistical significance (*P*<0.05). When studies adjusted their results for multiple testing, we used the p-value threshold after the adjustment to define statistical significance, otherwise we used nominal significance level (*P*<0.05). When a p-value was not reported for the main MR estimate, we calculated it using the effect size and standard error. Also, when studies also reported analyses excluding genetic variants with evidence of pleiotropy, we considered those as the main analysis as they better account for the MR assumptions. The term “robust” refers to evidence of causality for the studied associations, not the quality of the analysis. An association was supported by *probable* evidence for causality when at least one method (main or sensitivity analysis) achieved statistical significance and the direction of the effect estimate was concordant in all methods. *Suggestive* evidence for causality was achieved when at least one method had a statistically significant p-value, but the direction of the effect estimates differed between methods. Associations that presented non-significant p-values for both the main analysis and sensitivity analyses were classified as *insufficient* evidence for causality. Polygenic MR studies which did not report any sensitivity analyses were *non-evaluable* based on the above grading scheme which focuses on evaluating the robustness for causality of the studied associations.

Monogenic MR studies included MR analyses examining single SNPs or single gene regions to define the risk factor (instrumental variable of interest).. Most of these studies could not preform sensitivity analyses as the number of genetic variants was small. We assessed the robustness of these results based on whether the authors perform also colocalization analysis[8]. Colocalization assesses whether the same genetic variant (or variants) influences two traits and is useful when MR is based on a single gene region [7].

Finally, we further assessed the reporting of all MR studies using the Strengthening the Reporting of Mendelian Randomization Studies (STROBE-MR) Guidelines [4, 5].

All statistical analyses were done with R 4.1.0.

## Results

### Eligible studies

The literature search yielded 3,980 papers of which 586 were evaluated in full text and, of them, 391 publications were deemed eligible (see full list in Supplementary Files S1 and S2 respectively). The majority of studies were published from 2018 onwards (Figure S2).

### Description of study characteristics

**Figure 2:**
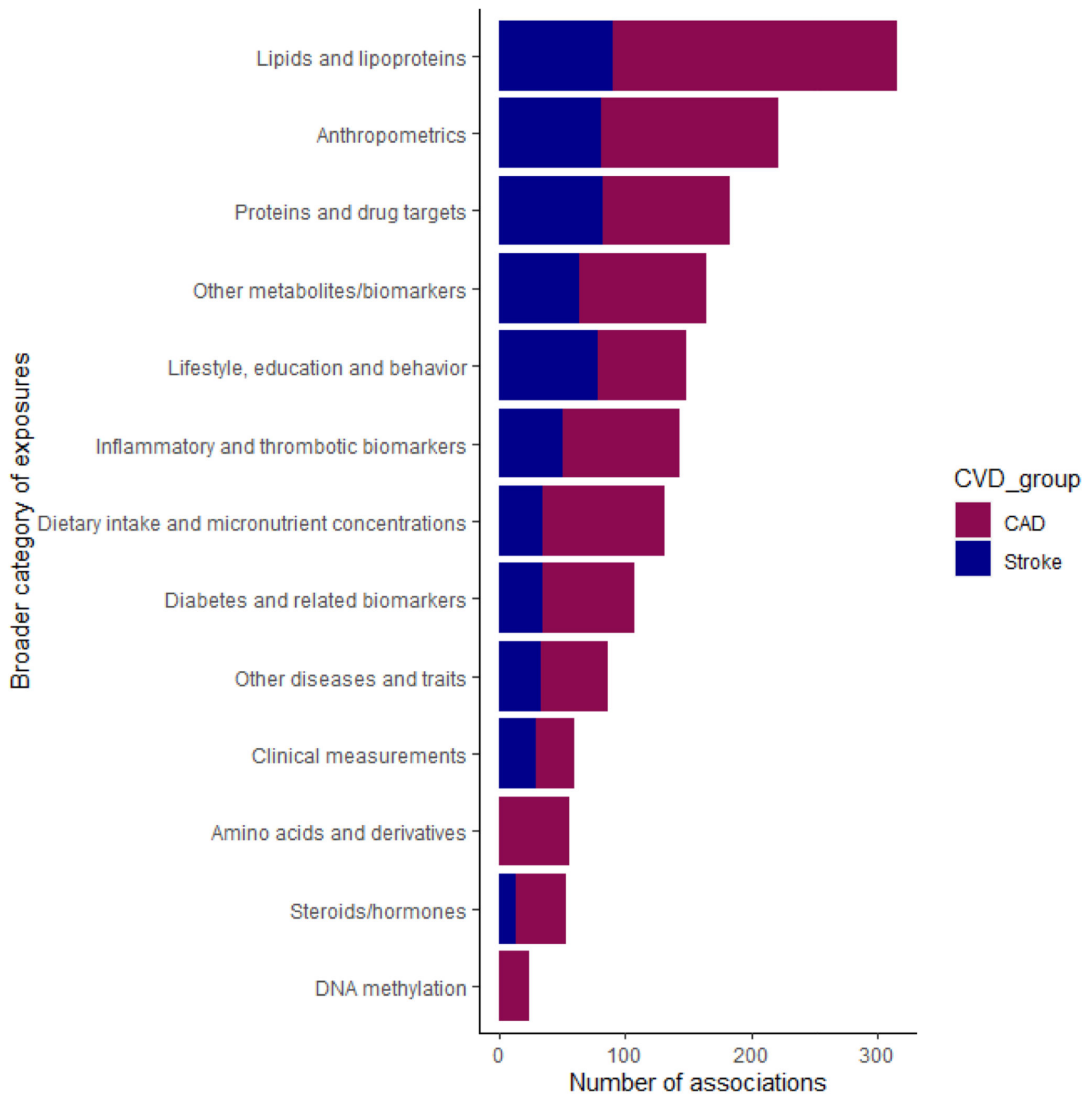
Number of Mendelian randomization (MR) associations extracted from eligible publications according to different exposure categories for coronary artery disease (CAD) and stroke.

Of 391 MR publications, 317 studied CAD as the outcome of interest, 175 stroke and 102 both outcomes. Overall, the 391 publications included 2,7 different MR analyses examining 535 unique exposures, 482 in relation to CAD and 268 in relation to stroke, covering a broad range of biomarkers, physical measurements, traits and diseases (Figure 2, Supplementary Table S1). Many risk factors have been examined in multiple MR publications (Supplementary Table S2) and the most commonly studied risk factors were LDL cholesterol for CAD (28 papers) and body mass index (BMI) for stroke (10 papers).

There were 2,122 polygenic MR analyses and 596 monogenic MR analyses. The median number of SNPs used to genetically predict the risk factor of interest in polygenic MR studies was 14, ranging from two to 3,188 SNPs (Supplementary Table S3). The median sample size for the exposure genetic analysis was 79,366 (with the smallest being 272 for phospholipase A2 and the largest 1,887,658 for COVID-19 severity. Genome-wide association study (GWAS) summary statistics for the exposures were derived from European (87.5%) and multi-ethnic (9.9%) ancestry populations. Regarding the outcome, 65.9% of the associations were derived from European and 32.5% from multi-ethnic populations. The vast majority of MR analyses used or included CARDIoGRAMplusC4D (Coronary ARtery DIsease Genome wide Replication and Meta-analysis (CARDIoGRAM) plus The Coronary Artery Disease (C4D) Genetics consortium) when CAD was the outcome of interest (822 out of 1,478 MR analyses) and MEGASTROKE when stroke was the outcome of interest (914 out of 1,214 MR analyses). Only 65 MR analyses were based on one sample MR designs.

### Evaluation of the robustness of causal associations

Supplementary Table S4 lists the main characteristics of each eligible MR analysis and its subsequent grading category based on the robustness of evidence, while supplementary Table S5 summarizes the grading categories. Out of the 2,122 polygenic MR publications, 20 analyses were based on two genetic variants (in different gene regions) and could not perform sensitivity analysis. From the remaining 2,102 MR analysis, 1,479 (70%) presented results on both the main and at least one sensitivity analysis and were eligible for evaluation. IVW was the main analysis in the majority of papers examined (N=1,931, 92%). Of the 1,479 associations reporting main and sensitivity analyses, we found 138 *robust* associations (median N_SNPs_=110), 346 *probable* associations (median N_SNPs_=71), 109 *suggestive* associations (median N_SNPs_=73) and 886 associations with *insufficient* information (median N_SNPs_=21).

Overall, 276 MR analyses reported multivariable MR (MVMR) which examines multiple risk factors (exposures) simultaneously and estimates the independent causal effect of each of the risk factors. Genetically predicted BMI, smoking and lipid levels were common risk factors adjusted in MVMR.

There were also 596 monogenic MR associations examining genetic variants within a single gene region as instrumental variables for the risk factor of interest. Of them, 447 were based on a single genetic variant analysis only (Supplementary Figure S3). Among the 596 associations, 219 reported statistically significant results in the main analysis. Of them, 24 analyses performed colocalization analyses with the outcome of interest, of which two found evidence for colocalization between the risk factor and the outcome (Islet cell autoantigen 1-like protein (ICA1L) for stroke and neurokinin 3 receptor (NK3R) for CHD).

**Figure 3:**
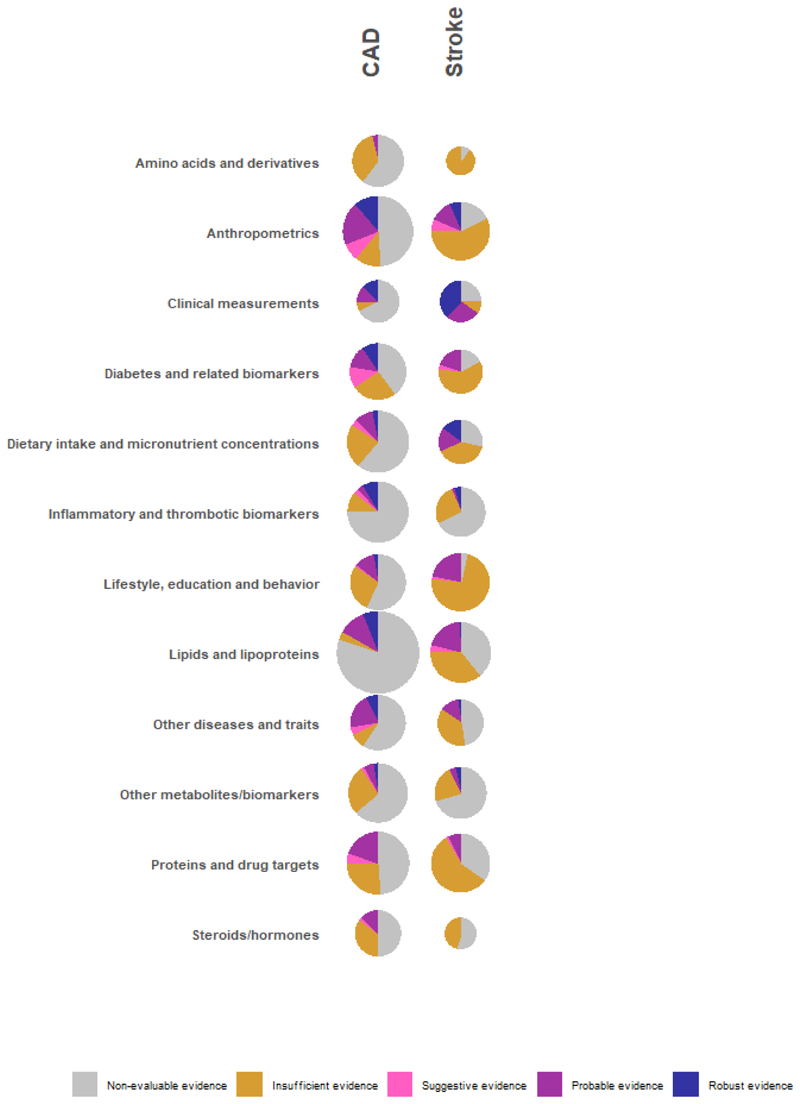
Evidence map for eligible Mendelian randomization (MR) studies per exposure category for coronary artery disease (CAD) and stroke. DNA methylation was not included in the diagram because of the limited number of analyses for the specific exposure category. Non-evaluable evidence level includes associations for which a sensitivity analysis was not feasible (e.g., single genetic variant analyses).

**Figure 4:**
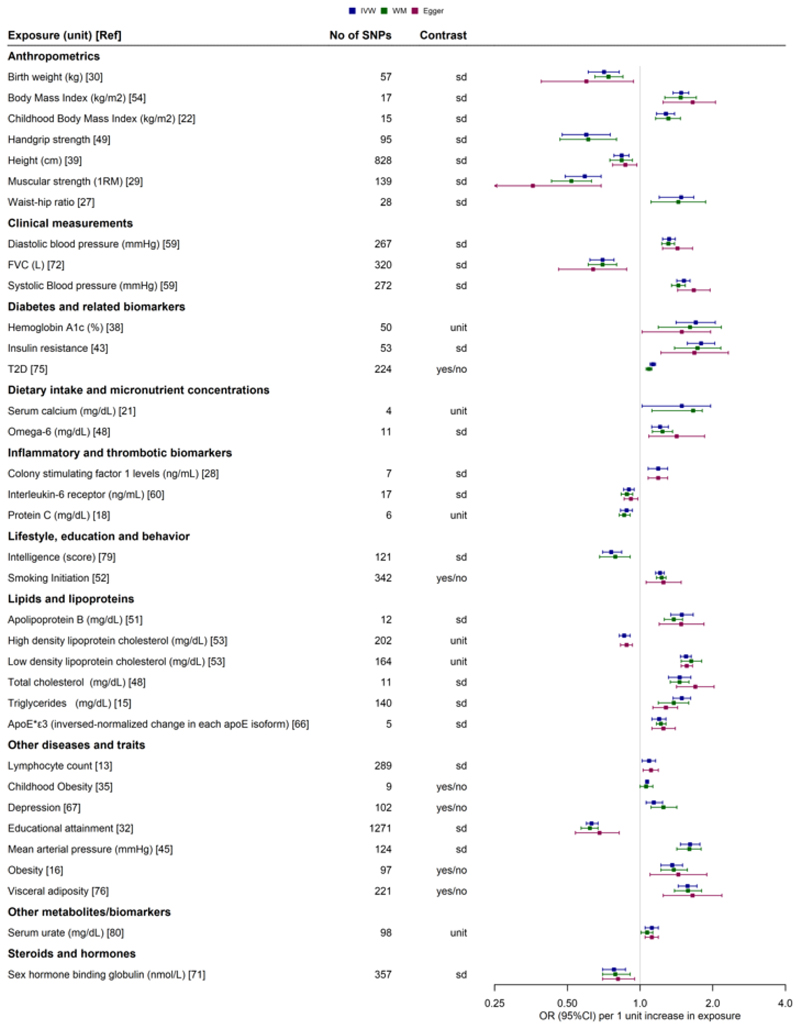

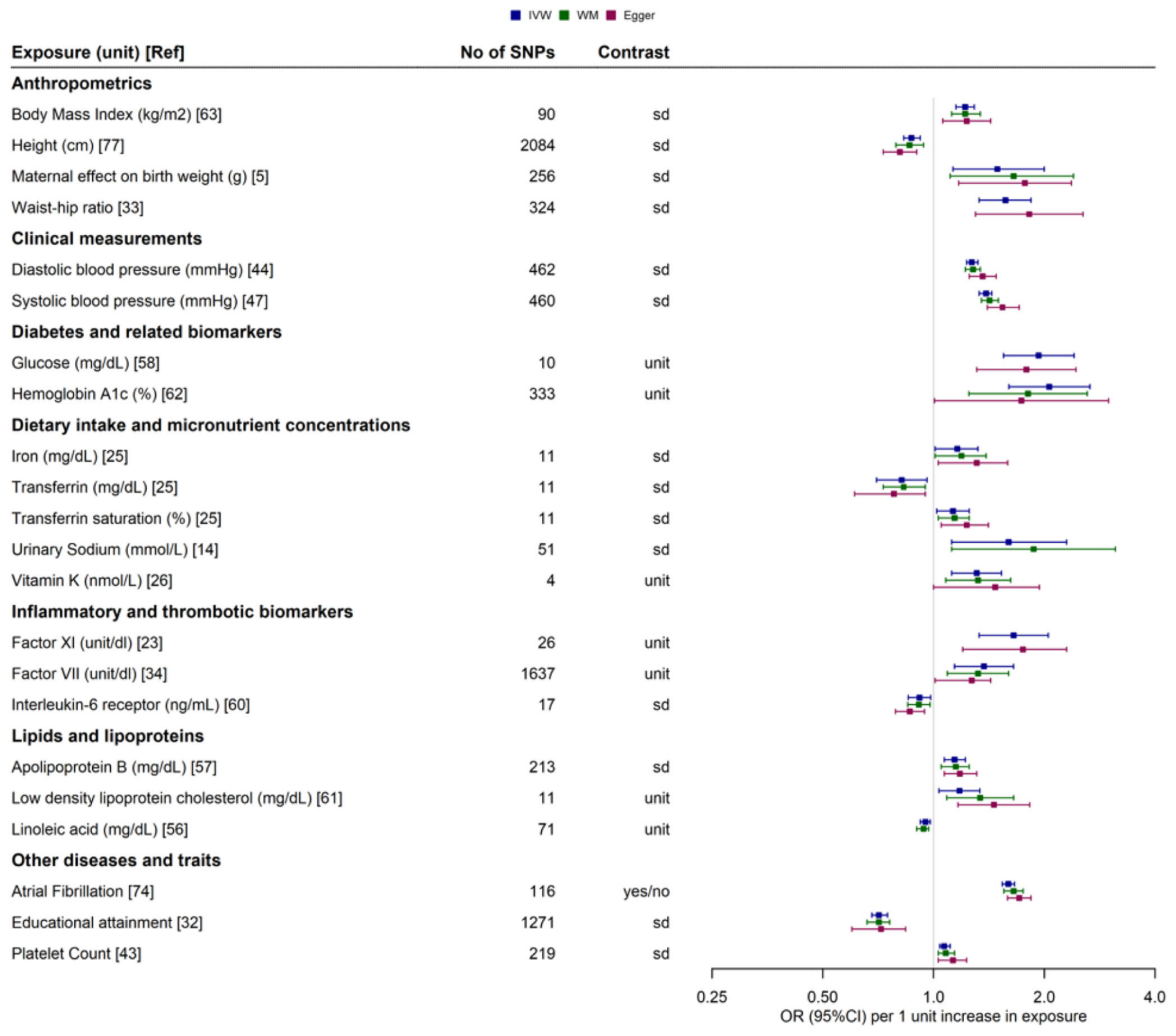
Forest plot showing the identified *robust* associations between exposures and coronary artery disease (CAD) (A) and stroke (B). When more than one study exhibited a *robust* association with coronary artery disease (CAD) or stroke, the most recent study with the largest sample size (exposure) was selected.

A graphical overview of the robustness of the evidence per exposure category and CVD group is presented in Figure 3. The exposure category with the most *robust* associations was anthropometry (N=28), followed by lipids and lipoproteins (N=24). There were 138 *robust* polygenic MR associations pertaining to 53 different risk factors as illustrated in detail in Supplementary Table S6 and Figure 4 (35 risk factors for CAD and 22 risk factors for stroke [9–76]. Almost all studies that showed robust evidence of association between an exposure and CAD or stroke had a good reporting score for MR-STROBE reporting, with the exception of three studies (Supplementary S7) [60, 71, 73]. Apart from conventional cardiovascular risk factors such as blood pressure traits, cholesterol levels, type 2 diabetes, obesity and smoking, *robust* positive associations were observed between genetically predicted calcium (IVW OR: 1.66, 95%CI: 1.12-1.81) lymphocyte count (IVW OR:1.09, 95%CI:1.02 - 1.16), colony stimulating factor 1 (IVW OR: 1.19, 95%CI: 1.08 - 1.30) and omega 6 fatty acid levels (IVW OR: 1.21, 95%CI: 1.12-1.31) with CAD. Protective (inverse) associations were also observed for genetically predicted height (IVW OR: 0.84, 95%CI:0.78 – 0.90), forced vital capacity (FVC) (IVW OR:0.64, 95%CI: 0.46 - 0.88), sex hormone binding globulin (IVW OR: 0.79, 95%CI: 0.7 - 0.91) and interleukin 6 receptor (IL6R) (IVW OR: 0.9, 95%CI:0.85 - 0.95). For stroke *robust* associations were observed for genetic predisposition to several thrombotic factors, apolipoprotein B (IVW OR: 1.14, 95%CI: 1.07 - 1.22) and urinary sodium excretion (IVW OR: 1.6, 95%CI: 1.12 - 2.3) (positive) and IL6R (IVW OR: 0.92, 95%CI: 0.85 - 0.98) and transferrin (IVW OR: 0.82, 95%CI: 0.70 - 0.96) (inverse).

Of the robust associations, 24 reported multivariable MR analyses adjusting for potential mediators. Of them, two associations were attenuated to the null; linoleic acid and stroke after adjusting for LDL cholesterol and FVC and CHD after adjusting for height. The rest of multivariable analyses attenuated the estimates showed different levels of mediation but statistical significance was retained.

## Discussion

In this systematic review, we summarized the evidence for associations between genetic predisposition to 535 risk factors and CAD or stroke examined in 391 publications covering 2,718 MR associations. Using a set of predefined criteria, we found *robust* evidence for causality between 35 distinct risk factors and CAD and between 22 risk factors and stroke. For CAD, these included the well-established cardiovascular risk factors such as blood pressure, type 2 diabetes, obesity, smoking and cholesterol levels but also, anthropometry and physical measurements (height, birth weight, muscular strength and FVC) and several biomarkers (leucocyte count, serum calcium, IL6R signaling, protein C, omega 6 fatty acid levels, sex hormone binding globulin). Stroke showed a somewhat different profile of associations, with evidence for causal effect for thrombotic risk factors (factor VII, factor XI, platelet count, vitamin K), iron and inflammatory biomarkers (iron, transferrin, transferrin saturation, IL6R), and blood pressure.

This large body of published MR analyses highlighted several reporting limitations also observed in a previous systematic review of MR studies on cancer outcomes [77]. Approximately half of the associations included sensitivity analyses, which are important to assess the assumptions of the method and therefore the robustness of the results. The lack of sensitivity analyses was often because studies were published early, prior to the availability of MR sensitivity methods, or because they were monogenic (single-gene) or single variant MR studies where sensitivity analyses were not feasible due to the small number of IVs. In the latter case of MR studies, colocalization can be used to investigate whether the exposure and the outcome share a causal variant in the genetic region but it was rarely performed in the examined MR studies. Again this may be due to the fact that colocalization was only suggested recently as a additional method to support monogenic MR investigations and a large proportion of these studies were published earlier. Genetic variants typically explain only a small proportion of the variation in the relevant exposure of interest and as a result, low statistical power is common in MR studies. MR studies examined here rarely reported power estimates and/or the variance explained by IVs and therefore it was difficult to conclude whether non-significant associations were true null findings [78]. The recent publication of MR reporting guidelines (STROBE-MR) statement should improve the reporting standards of MR studies and further enhance the robustness and interpretability of MR findings [5]. Finally, MR investigations are dependent on the primary GWAS sources the quality of which was not assessed in this work. However, underlying GWAS quality are unlikely to lead to false positive results.

A considerable proportion of the studies provided supporting evidence for causal associations between the so-called conventional CVD risk factors and CVD events. We identified studies with *robust* evidence for causal associations between genetically predicted LDL cholesterol, HDL cholesterol, triglycerides, apolipoprotein B, blood pressure, type 2 diabetes, and glycemic traits such as HbA1c and insulin resistance with CAD and stroke. This supports the extensive evidence from traditional epidemiological studies, experimental studies, and randomized control trials examining these risk factors. However, MR provided additional valuable information such as examination of comparative effects between correlated risk factors, estimation of non-linear effects and interactions with other factors. For example, multivariable MR analyses on several lipids and lipoproteins highlighted the central role of apolipoprotein B compared to other lipids in ischemic stroke [53]. Similarly, the MR paradigm generated evidence supporting an effect of midlife blood pressure on later life CAD risk independent of later life blood pressure [41].

Measures of anthropometry have also been extensively studied in the MR context in relation to CAD and stroke. Beyond BMI, which showed *robust* causal associations with CAD and stroke, higher height was also highlighted as a potentially causal risk factor for CAD. Genetically predicted height also mediated at least of the association between lung function measured by FVC and CHD and stroke. Although several observational studies have reported a protective role of short height for CVD, the magnitude of this association has been controversial [79] and the mechanisms underlying this inverse association are not well understood [80]. One proposed explanation is that shorter individuals have on average smaller vessel diameter, which can lead to increased arterial occlusive events [81, 82]. There was also both *robust* and *probable* evidence for a protective association between higher birthweight and CAD and stroke respectively supporting the fetal developmental origins of CVD [26, 83].

Interestingly, further investigation into the fetal and/or maternal components of instrumental effects on birth weight showed *robust* evidence between lower birthweight, by maternal rather than fetal genome, and stroke and its subtypes in later life [51].

Lifestyle is an important area of MR research in CVD as RCTs are often inappropriate or unfeasible and evidence stemming from MR is vital to support causality. Smoking behavior showed *robust* evidence for causal association with CVD in agreement with overwhelming evidence from observational epidemiology [84]. Coffee, alcohol consumption and sleep duration also showed *probable* associations [48, 85–90]. Observational epidemiology has often suggested possible protective effect of moderate alcohol consumption on CVD. Dose response MR analyses did not support this conclusion and they found evidence of a dose–response relationship between alcohol and risk of stroke [91]. Educational attainment was reported to have a protective role for both CAD and stroke, exhibiting *robust* evidence of association in MR studies [15, 28, 33]. Traditional observational studies and MR mediation analyses have shown that BMI, systolic blood pressure, and smoking behavior mediate a substantial proportion of the protective effect of education on the risk of CVD outcomes [92, 93]. Despite the research interest on diet and CVD, there were few *robust* or *probable* associations between nutrients or dietary traits and CVD outcomes. This is in partly expected due to the weak genetic instruments on some nutrients and other dietary variables (few SNPs available to instrument dietary traits) and the small heritable components of many dietary traits both leading to under-powered MR studies.

Many MR analyses concentrate on the causal association between biomarker levels and CVD to identify novel treatment targets for the disease. Of them, genetically-predicted plasma soluble IL6 receptor (sIL6R), an IL6 signaling biomarker, showed *robust* evidence for an inverse association with CAD [36] and stroke [60] supporting a key role of inflammation in CVD which has also supportive RCT evidence [94]. Thrombotic factors are implicated in the coagulation cascade and along with inflammatory factors are contributing to the suppression of a pathogen entering in the host, a mechanism termed as immunothrombosis. The aberrant activation of immunothrombosis has been associated with increased risk for myocardial infarction, stroke and venous thromboembolism [95]. This association is supported by MR evidence. A *robust* positive association was observed between vitamin K and large vessel stroke as well as between two enzymes of the coagulation cascade (i.e., factor XI and factor VII) and ischemic stroke [19, 22, 30]. In contrast to thrombotic factors, Protein C, also known as factor XIX, is a zymogen that inactivates thrombotic enzymes and showed evidence for an inverse causal effect with CAD [14]. In concordance with meta-analyses of RCTs for calcium supplementation [96, 97], MR evidence supported a causal association between higher serum calcium levels and increased CAD risk [17, 98–100]. Circulating calcium levels are thought to increase CAD risk through vascular calcification [101, 102] or via the upregulation of the coagulation pathway which in turn is associated with CVD risk [103]. Finally, iron, ferritin, and transferrin saturation, biomarkers of iron metabolism and intake [104], showed *robust* positive causal associations with risk of stroke in MR analyses and this effect was suggested to be driven by an increased risk of cardioembolic stroke [21]. The latter, along with the absence of an association with CAD, may indicate that the effect of iron on stroke is through thrombus formation rather than atherosclerosis [105].

## Strengths and limitations

In the current systematic review, we summarized all previously published MR studies for all genetically determined exposures and their association with CVD risk. A clear categorization scheme and evaluation criteria were applied, to further examine the robustness credibility of the resulted associations. Other efforts to summarize the evidence of MR analyses on CVD risk have been performed in the past. However, they were either limited to specific exposures [106], or used a more narrative approach of presenting and assessing the MR results [107], while none performed a formal evaluation of the evidence. However, some limitations exist, which need to be acknowledged. Some relevant MR studies may have been missed through our search strategy, especially if the MR analysis was not the primary focus but only a supplementary analysis, which seems to be increasingly common in recent GWAS. In the absence of comprehensive MR guidelines we based our evaluation of the evidence of causality adapting a set of previously proposed criteria. This approach did not allow us to investigate MR studies presenting only main analysis without sensitivity analyses. Sensitivity analyses increase the credibility of the findings as they test various MR assumptions. However, many studies did not present those as they were published earlier before such analyses were introduced in the literature or were based on monogenic associations with a small number of SNPs which did not allow sensitivity analyses. For the later associations we based our evaluation on availability of colocalization analysis which was again introduced only recently. Therefore the evaluation criteria for this systematic review were designed mainly for the assessment of the evidence that resulted from the MR analyses and not for the assessment of the quality of the studies. Although many studies included instrumental variables from the largest available GWAS for the exposure traits, the SNPs explained a small percentage of the variance and therefore some studies were underpowered. [108]. Finally, information on statistical power of the instrument was often not reported, and therefore the grading scheme used could not distinguish between MR analyses with robust evidence of lack of association or MR analyses which did not present an association due to lack of power.

## Conclusions

MR studies have contributed a large body of evidence into the causal association between risk factors and CVD. Although many studies concentrated on CVD risk factors known to be causally associated to CVD through RCTs, MR provided confirmation of previous associations, supported evidence for potentially novel causal risk factors as well as refuted several associations suggested by observational studies. Despite the plethora of MR investigations in CVD, the highlighted associations with *robust* evidence for causality were modest. Those risk factors concentrated around conventional risk factors for CVD, inflammation and thrombotic factors and indices of anthropometry and showed a large overlap between risk factors for CAD and stroke as well as highlighted the different risk factor profiles between stroke subtypes. As GWAS investigations of exposures become larger, novel exposures are measured in epidemiological settings, and novel MR methodologies are published, the contribution of MR in establishing causal associations and prioritizing novel RCT is expected to grow further.

## Supplementary Material

Supplementary material
