# Supplementary material for: Appraising the Causal Role of Risk Factors in Coronary Artery Disease and Stroke: A Systematic Review of Mendelian Randomization Studies
Source: J Am Heart Assoc. 2023 Oct 17;12(20):e029040. doi: 10.1161/JAHA.122.029040 (PMC7615320; doi:10.1161/JAHA.122.029040)
Supplement: Supplementary file 1 [file JAH3-12-e029040-s002.pdf]

SUPPLEMENTAL MATERIAL

Supplemental Methods

Data S1: Inclusion-Exclusion criteria

MR studies that evaluated the association of any genetically predicted risk factor with risk of coronary heart disease or stroke were deemed eligible. We only considered full original publications, and conference abstracts, letters, commentaries, editorials, reviews, study proposals and theoretical papers were excluded. We also excluded: a) studies that did not state that MR or a genetic instrumental variable was used and b) studies that only evaluated marginal genetic associations, namely direct associations of the genetic IVs with cancer without incorporating the association of the IVs with the risk factors. Methodological studies using an application of MR only as an example, and studies that identified potential genetic instruments for future MR studies were also deemed ineligible. Finally, we excluded studies that did not provide usable data, namely at least an effect size for the studied association and a corresponding measure of uncertainty.

Data S2: Data Extraction

From all the eligible studies the following information was retrieved: name of first author, year of publication, risk factor evaluated, type of CVD, number of subjects and number of cases for both the exposure and the outcome analyses, source of exposure and outcome populations, population ancestry (for both the exposure and the outcome analyses), number of genetic instruments, exclusion criteria applied for the genetic instruments (p-value threshold, threshold for linkage disequilibrium, biological relevance), power of the genetic instruments and the corresponding effect estimate (when available), percentage of variance explained by the instruments, MR design (one-sample or two-sample, based on whether the gene-exposure and gene-outcome associations were estimated on the same or different populations), the specific analytical approach used for the main analysis as defined by the original publication authors (if multiple analyses were presented but it was unclear which was the main MR analysis, we opted for the inverse-variance-weighted analyses, if available), unit of comparison, the effect metric assessed (Odds Ratio, Hazard Ratio, beta, etc.), the effect size and the corresponding 95% confidence interval (CI) and p-value, the Q-statistic for heterogeneity for the instrument and the accompanying I2 value. Since studies frequently assessed multiple risk factors and/or CVD outcomes, we also extracted information on any adjusted thresholds used for multiple testing and whether the reported associations survived these thresholds. We further extracted information on a number of sensitivity MR methods, whenever these were performed and reported, namely MR-Egger, weighted median (WM), MRPRESSO and multivariable MR (MVMR).

MR-Egger is useful for the detection of IV assumption violation and consists of three parts: (1) a test for directional pleiotropy, (2) a test for a causal effect, and (3) an estimate of the causal effect [5].

Weighted median method provides a consistent estimate of the causal effect, assuming that most genetic variants are valid IVs [6]. MR-PRESSO evaluates horizontal pleiotropy in multi-instrument by combining the following three components (Methods and Fig. 1): (a) detection of horizontal pleiotropy (the MR-PRESSO global test); (b) correction for horizontal pleiotropy via outlier removal (the MR-PRESSO outlier test); and (c) testing of significant differences in the causal estimates before and after correction for outliers (the MR-PRESSO distortion test) [7].

Multivariable MR (MVMR) is an extension of Mendelian randomization that uses multiple genetic variants associated with several measured risk factors to simultaneously estimate the causal effect of each of the risk factors on the outcome. MVMR can be beneficial when genetic variants are associated with several related risk factors [8].

Data S3: The grading was performed in the following manner:

The associations were considered as having Robust evidence for causality when both the main analysis and at least one sensitivity analysis achieved significance (when multiple testing was reported, we used the p-value reposted after the adjustment, otherwise we used nominal significance level). Furthermore, in all methods the direction of the effect estimates needed to be concordant. Probable evidence for causality was graded when at least one method (main or sensitivity analysis) achieved significance and direction of the effect estimate was concordant for all the methods. Suggestive evidence for causality was achieved when at least one method had a significant p-value but the direction of the effect estimates differed between methods. Associations that presented non-significant p-value for both the main analysis and sensitivity analyses were classified as insufficient evidence for causality. Lastly, associations that did not present any of the sensitivity analyses, were categorized as non-evaluable evidence.

Table S7: Number and percent of Mendelian randomization analyses per grading category by CVD group

	Robust	Probable	Suggestive	Insufficient	Non-evaluable
All data	141 (5.17%)	353 (12.95%)	110 (4.04%)	926 (33.98%)	1,195 (43.85%)
CHD	89 (5.89%)	178 (11.78%)	68 (4.50%)	316 (20.91%)	860 (56.92%)
Stroke	52 (4.28%)	175 (14.42%)	42 (3.46%)	610 (50.25%)	335 (27.59%)

Table S10: Robust associations being downgraded to probable after adjusting for a possible confounder.

Author, year	PMID	Exposure	Broader category of exposures	CVD	CVD group	MVMR adjustment	MVMR p-value	Evaluation
Ye Ding et al., 2021	33584789	Linoleic acid	Lipids, lipoproteins and derivatives	Ischemic Stroke	Stroke	LDL-C	0.13	Probable
	33584789	Linoleic acid	Lipids, lipoproteins and derivatives	Large artery stroke	Stroke	LDL-C	0.11	Probable
Gill et al., 2021	34002035	BMI	Anthropometrics	Stroke	Stroke	Systolic Blood Pressure, Diabetes, Lipids, Smoking	0.28	Probable
	34002035	WHR	Anthropometrics	Coronary artery disease	CHD	Systolic Blood Pressure, Diabetes, Lipids, Smoking	0.15	Probable
Chan et al., 2021	34750372	DBP	Clinical measurements	Coronary artery disease	CHD	SBP	0.03	Probable
	34750372	DBP	Clinical measurements	Coronary artery disease	CHD	SBP	0.26	Probable
	34750372	DBP	Clinical measurements	Ischemic stroke	Stroke	SBP	0.95	Suggestive
	34750372	DBP	Clinical measurements	Coronary artery disease	CHD	SBP	0.07	Probable
	34155093	FVC	Clinical measurements	Coronary artery disease	CHD	height	0.22	Probable

Figure S1. Categorization of the evidence.

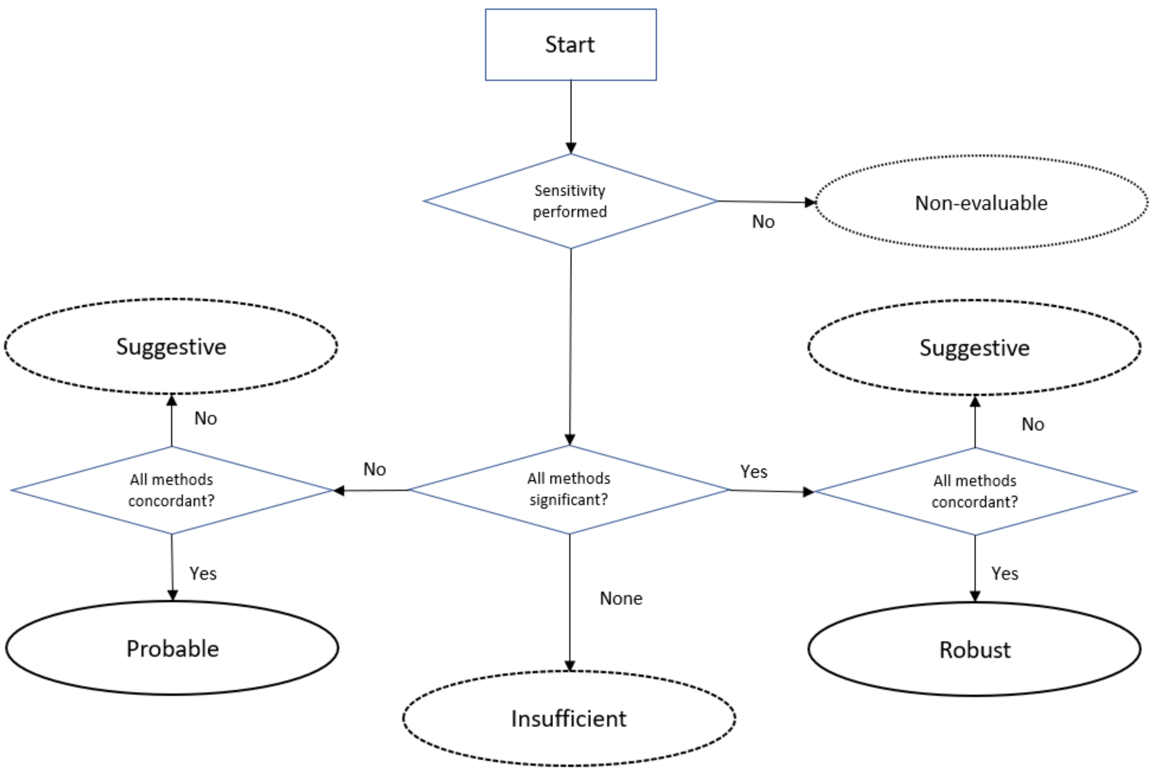

For the main analysis: statistically significant at the threshold set up by the study due to multiple testing or at 0.05 if no multiple testing threshold was defined. For the sensitivity analyses: statistically significant at 0.05

Figure S2. Frequency of MR publications on CVDs from 2005-2022.

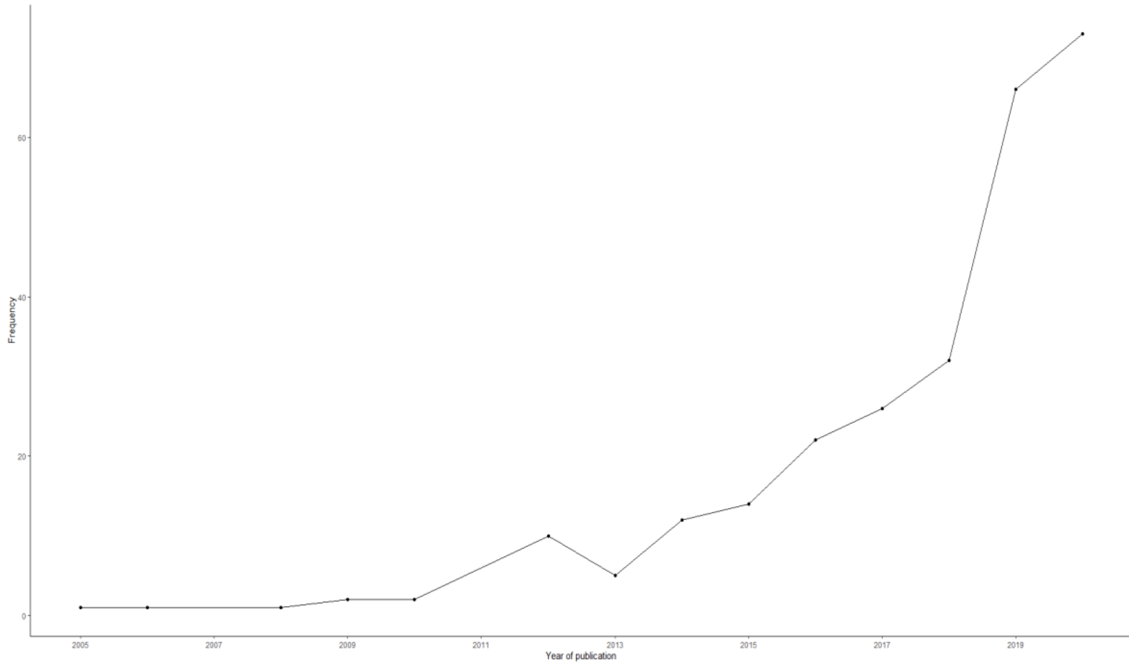

MR: Mendelian randomization, CVDs: Cardiovascular diseases

Figure S3A

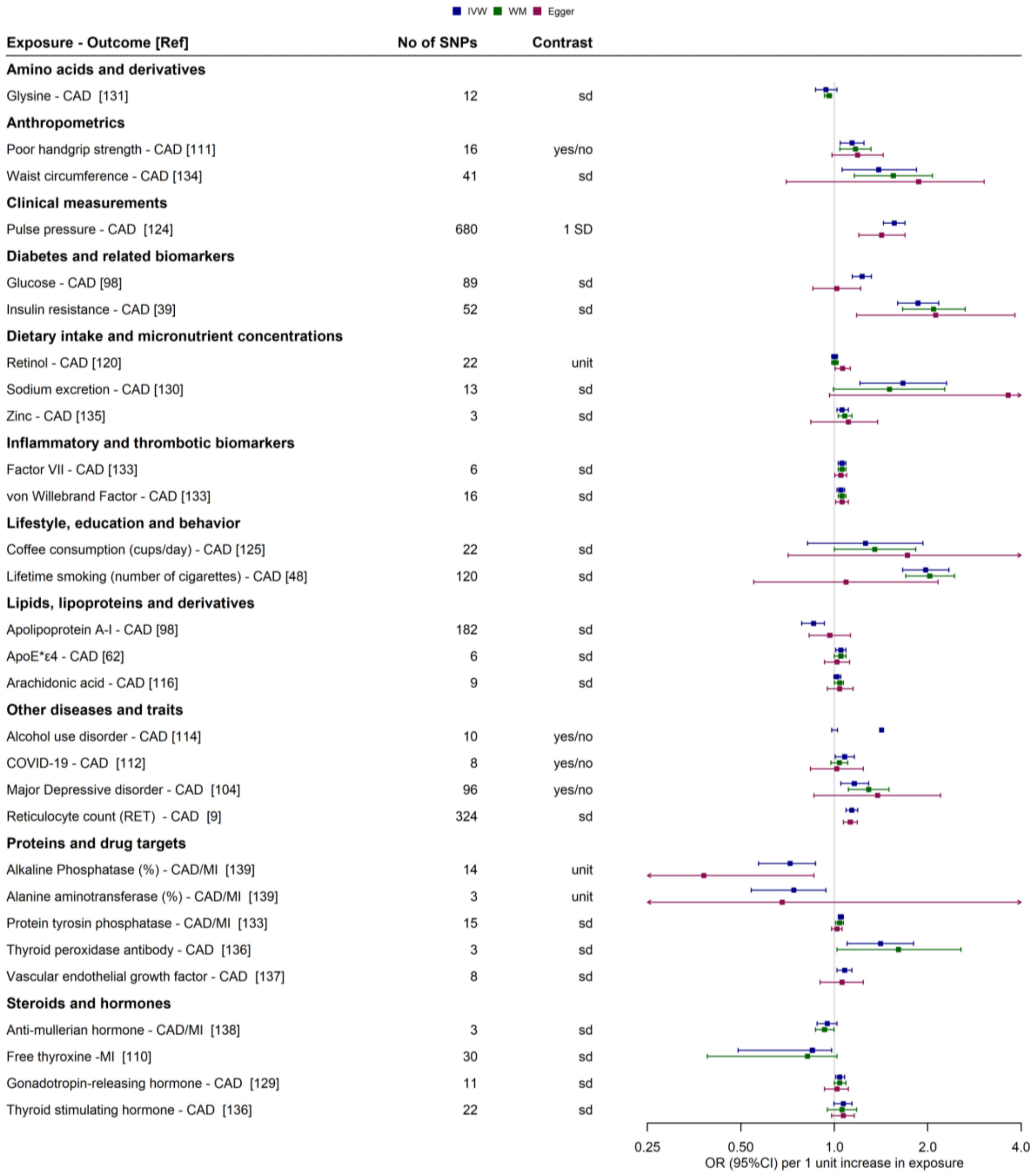
IVW WM Egger
Exposure - Outcome [Ref]
No of SNPs
Contrast
Amino acids and derivatives
Glysine - CAD [131] 12 sd
Anthropometrics
Poor handgrip strength - CAD [111] 16 yes/no
Waist circumference - CAD [134] 41 sd
Clinical measurements
Pulse pressure - CAD [124] 680 1 SD
Diabetes and related biomarkers
Glucose - CAD [98] 89 sd
Insulin resistance - CAD [39] 52 sd
Dietary intake and micronutrient concentrations
Retinol - CAD [120] 22 unit
Sodium excretion - CAD [130] 13 sd
Zinc - CAD [135] 3 sd
Inflammatory and thrombotic biomarkers
Factor VII - CAD [133] 6 sd
von Willebrand Factor - CAD [133] 16 sd
Lifestyle, education and behavior
Coffee consumption (cups/day) - CAD [125] 22 sd
Lifetime smoking (number of cigarettes) - CAD [48] 120 sd
Lipids, lipoproteins and derivatives
Apolipoprotein A-I - CAD [98] 182 sd
ApoE*ε4 - CAD [62] 6 sd
Arachidonic acid - CAD [116] 9 sd
Other diseases and traits
Alcohol use disorder - CAD [114] 10 yes/no
COVID-19 - CAD [112] 8 yes/no
Major Depressive disorder - CAD [104] 96 yes/no
Reticulocyte count (RET) - CAD [9] 324 sd
Proteins and drug targets
Alkaline Phosphatase (%) - CAD/MI [139] 14 unit
Alanine aminotransferase (%) - CAD/MI [139] 3 unit
Protein tyrosin phosphatase - CAD/MI [133] 15 sd
Thyroid peroxidase antibody - CAD [136] 3 sd
Vascular endothelial growth factor - CAD [137] 8 sd
Steroids and hormones
Anti-mullerian hormone - CAD/MI [138] 3 sd
Free thyroxine -MI [110] 30 sd
Gonadotropin-releasing hormone - CAD [129] 11 sd
Thyroid stimulating hormone - CAD [136] 22 sd
0.25 0.50 1.0 2.0 4.0
OR (95%CI) per 1 unit increase in exposure

Figure S3B

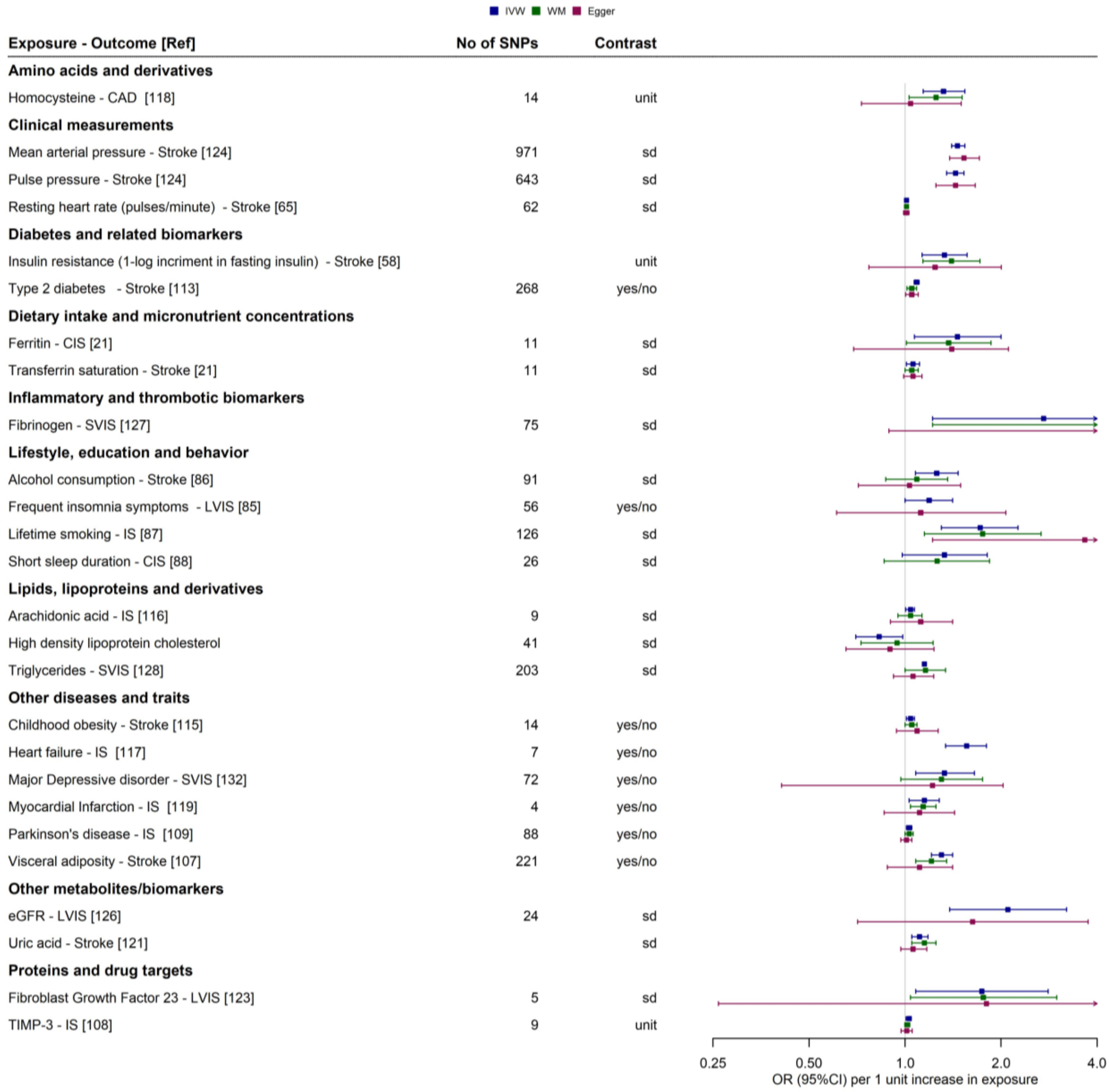

Figure S3. Forest plot showing the identified probable associations between exposures CAD (A) and stroke (B). When more than one study exhibited a probable association with CAD or stroke, the most recent study with the largest sample size (exposure) was selected. Abbreviations: CAD: coronary artery disease, MI: Myocardial infarction, IS: Ischemic stroke; LVIS: Large vessel ischemic stroke, SVIS: Small vessel ischemic stroke, CIS: Cardioembolic ischemic stroke, Ref: Reference [9, 21, 48, 58, 62, 85-88, 98, 104, 107-139].
